# The impact of generative AI on health professional education: A systematic review in the context of student learning

**DOI:** 10.1111/medu.15746

**Published:** 2025-06-18

**Authors:** Thai Duong Pham, Nilushi Karunaratne, Betty Exintaris, Danny Liu, Travis Lay, Elizabeth Yuriev, Angelina Lim

**Affiliations:** ^1^ Faculty of Pharmacy and Pharmaceutical Sciences Monash University Parkville VIC Australia; ^2^ Office of the Deputy Vice‐Chancellor (Education) The University of Sydney Camperdown NSW Australia; ^3^ Murdoch Childrens Research Institute Royal Children's Hospital Parkville VIC Australia

## Abstract

**Background:**

Generative Artificial Intelligence (GenAI) is increasingly integrated into health professions education (HPE), offering new opportunities for student learning. However, current research lacks a comprehensive understanding of how HPE students actually use GenAI in practice. Laurillard's Conversational Framework outlines six learning types—*acquisition, inquiry, practice, production, discussion and collaboration*—commonly used to categorise learning activities supported by conventional and digital technologies. Gaining insight into how GenAI aligns with these six learning types could assist HPE academics in integrating it more thoughtfully and effectively into teaching and learning.

**Purpose:**

This systematic review investigates how HPE students utilise GenAI and examines how these uses align with Laurillard's six learning types compared to conventional and digital technologies.

**Material and Methods:**

A systematic review searching five major databases—*ERIC, Education Database, Ovid Medline, Ovid Embase and Scopus* including articles on HPE students' use of GenAI until 15th September 2024. Studies were included if they were conducted within formal HPE training programs in HPE and specifically mentioned how students interact with GenAI. Data were mapped to the six learning modes of the Laurillard's Framework. Study quality was assessed using the Medical Education Research Study Quality Instrument (MERSQI).

**Results:**

Thirty‐three studies met inclusion criteria. GenAI supported learning most frequently in *practice* (73%), *inquiry* (70%), *production* (67%) and *acquisition* (55%). These studies highlight GenAI's varied educational applications, from clarifying complex concepts to simulating clinical scenarios and generating practice materials. *Discussion* and *collaboration* were less represented (12% each), suggesting a shift toward more individualised learning with GenAI. The findings highlight benefits such as efficiency and accessibility, alongside concerns about critical thinking, academic integrity and reduced peer interaction.

**Conclusion:**

This review has provided insights into HPE students' learning aligned with Laurillard's existing six learning types. Although GenAI supports personalised and self‐directed learning, its role in collaborative modes is under‐explored.

## Abstract 2

Using Laurillard's six types of learning, Pham et al. offer insights into how GenAI is already influencing health professional education, highlighting that its role in collaborative learning is under‐explored.

## 1 INTRODUCTION

Generative Artificial Intelligence (GenAI) has been integrated into healthcare, improving tasks such as clinical documentation, drug discovery and diagnostics, although challenges such as data accuracy and privacy persist.^1^ Changes are also occurring in health professional education (HPE), with GenAI offering new, transformative ways for learners to engage with technology that mimics human‐like responses.^2^, ^3^ GenAI offers exciting possibilities for education, but its integration also comes with significant challenges. Research indicates that overreliance on GenAI tools can hinder the development of critical skills essential for HPE students such as problem‐solving, writing and analytical reasoning.^4^ Moreover, GenAI poses risks to genuine learning, as many students use it without proper guidance, leading to unintentional cheating, misinformation and over‐reliance.^5^ This over‐reliance on GenAI may reduce students' ability to engage deeply with material, critically evaluate information and retain knowledge, which can impair their ability to perform in real‐world clinical settings and the ability to maintain the highest ethical and professional standards to qualify as competent health professionals.^6^, ^7^ The importance of understanding how students interact with GenAI cannot be overstated, as these interactions directly shape learning outcomes, an increasingly important metric in HPE.^8^, ^9^ It remains unclear whether these risks or challenges are currently affecting HPE student learning. Feigerlova et al^10^ similarly states that the current evidence on the educational outcomes of GenAI‐powered interventions in HPE is limited, with findings primarily based on small‐scale, single‐centre studies of short duration. Although there are reviews on how students use GenAI, existing reviews lack a specific focus on how GenAI impacts different types of learning activity. Utilising a well‐known learning activities framework like the Laurillard Conversational Framework that showcases six different learning types across conventional and digital technologies will be able to provide a more comprehensive and theoretically grounded understanding of how and where GenAI has impacted student learning.

Technology is increasingly integrated into various aspects of health professions education (HPE), with ongoing research exploring its impact.^11^ The transformative potential of learning technologies lies in their ability to enhance teaching and learning processes, ultimately leading to better educational outcomes. In this context, the Laurillard Conversational Framework has been recommended as a model for categorising technology‐enabled learning activities in HPE.^12^ Students' use of conventional and digital technology has been traditionally summarised by Laurillard's conversational framework^13^ which proposes six types of learning modes: 1) acquisition, 2) inquiry, 3) practice, 4) production, 5) discussion and 6) collaboration. Although *acquisition*, *inquiry*, *practice* and *production* are centred on personal learning experiences, *discussion* and *collaboration* rely on peer interaction, making them forms of social learning.^13^ This framework has been widely accepted for over a decade as a valuable tool for designing effective learning environments and activities.^14^, ^15^ By structuring learning in this way, educators can effectively design technology‐based activities that align with pedagogical goals and enhance student engagement.^12^ This framework has been used to the use of a digital anatomy learning platform in medical students,^16^ and has been suggested as a means of unpack findings that explore factors influencing the implementation, adoption, use, sustainability and scalability of mobile learning for medical and nursing education.^17^ Although it has not been widely used in HPE, it can provide exploration into how different learning environments impact the learner and their interactions, such as common learning modes of team‐based, self‐directed and context‐based learning and particularly unpack engagement with technology‐based tools which HPE have embraced such as elearning and mobile learning.^12^ GenAI presents another dimension to technology as its training enables it to generate human‐like, coherent, contextually relevant responses and at larger scales.^2^, ^18^, ^19^ This platform has been shown to give students a range of opportunities from facilitating practice, being a learning partner to bounce ideas off and also improving efficiencies in material generation.^20^, ^21^, ^22^ GenAI has disrupted traditional educational approaches, necessitating their reassessment to ensure effective and responsible use of GenAI.^23^ This review is important as it explores how GenAI may be encouraging more active forms of learning among students, reducing reliance on teachers and reshaping classroom dynamics, challenging traditional learning structures.^24^ Recent advancements offer enhanced tools for educational applications, particularly in content generation and multimodal learning^25^; however, there remains a significant gap in understanding how to productively and responsibly incorporate GenAI into HPE practices.^26^ Understanding how students engage with GenAI using this framework will be the first step to exploring its impact on student learning and highlighting what educators need to do to guide students. It will also highlight when GenAI is used the most or underutilised to help educators understand where to direct their efforts. The research question guiding this review is: *How do health profession students use GenAI, and how does this use align with the six learning types across Laurillard's Conversational Framework in comparison to conventional and digital learning tools?*

## 2 METHODS

### 2.1 Literature searching and screening

This systematic literature review was conducted in accordance with the 2020 Preferred Reporting Items for Systematic Reviews and Meta‐Analyses (PRISMA) guidelines.^27^ The protocol of the review is registered (PROSPERO CRD42024543726). This systematic review included original peer‐reviewed research papers that focussed on the application of GenAI technologies within formal HPE training programs within higher education. Grey literature was not included, as the focus was on peer‐reviewed studies to ensure reliability and consistency in the findings. Health Professionals were defined as those listed in the World Health Organisation classifying health workers document^28^ but refined based on the teams' perspectives of which professions had tertiary courses leading to an award of a university degree. No starting year limit was set, all studies published on or before 15th September 2024 were included. Studies were only included if they investigated student use of GenAI.

A comprehensive literature search was conducted across five major databases: ERIC, Education Database, OVID Medline, OVID Embase and Scopus. The database searches involved two stages using a combination of keywords, Medical Subject Headings (MeSH) and/or CINAHL subject headings (Appendix S1). In the first stage, search strings targeted GenAI applications and associated terminology, including terms such as ‘generative artificial intelligence’, ‘large language models’, ‘natural language processing’ and specific tools like ‘ChatGPT’, ‘Copilot’ and ‘Gemini’. The second stage focused on health professions education. The outcomes of both search strategies were collectively evaluated. All search terms are available in Appendix S1 and a detailed summary of eligibility criteria is included in Appendix S2. The screening and data extraction processes for this systematic review were managed using Covidence systematic review software, (Veritas Health Innovation, Melbourne, Australia). Two authors (TP and TL) independently assessed each study's relevance during the title and abstract screening phase. Author DL managed conflicts before the full‐text screening phase. The same process was applied for full text screening.

### 2.2 Study appraisal

The study quality was appraised using the Medical Education Research Study Quality Instrument (MERSQI)^26^ but no articles were excluded from the appraisal due to wanting to capture the scope of all GenAI use.

### 2.3 Synthesising data

The included papers were coded deductively using content analysis and mapped with Laurillard's six learning modes (acquisition, inquiry, practice, production, discussion and collaboration). The following definitions of the learning modes were applied: **Acquisition** defined as students receiving information through GenAI focusing on taking in knowledge alone; **Inquiry** defined as students actively exploring questions, gathering and analysing data through GenAI; **Practice** defined as actions that were repeated and accompanied with feedback cycles; **Production** defined as students working individually to create artefacts, such as presentations or essays; **Discussion** involves students engaging in conversations, sharing ideas with GenAI; **Collaboration** is defined as students working with another human peer on tasks or projects and using GenAI to facilitate team interaction and output. Coding was conducted independently by five team members (TP, AL, DL, NK and BE) then cross‐checked in a round table meeting with all authors. Each GenAI use was given its own definition, developed based on the recurrent patterns observed and the way GenAI was used in each study. The definitions of each learning action are discussed in Appendix S3. This process ensured that each GenAI use was categorised according to the nature of the activity and the purpose behind its use, allowing for a more precise alignment with the appropriate learning mode (acquisition, inquiry, practice, production, discussion and collaboration). Once the GenAI uses and their learning mode alignments were confirmed, five team members (TP, AL, DL, NK and BE) met to review the agreed‐upon framework and re‐examine the papers, ensuring consistency in matching the identified GenAI uses to their respective learning modes. Any discrepancies in the analysis were resolved through discussion and consensus among the team members.

## 3 RESULTS

### 3.1 Summary of studies

PRISMA diagram (Appendix S4) illustrates the study screening process. The initial database searches captured 1924 papers. Following the removal of the duplicates and the application of the inclusion and exclusion criteria, 33 papers met the eligibility criteria and were included in the final review. Appendix S5 describes the final 33 papers included in the review. Out of the 33 papers, 28 papers were published in 2024 highlighting the emerging growth of GenAI in education. The mean MERSQI score is 10.4 (range 1–18), with the MERSQI scores provided in Appendix S5. The average MERSQI score suggests the need for more high‐quality experimental and longitudinal studies to provide stronger evidence on how GenAI supports different learning modes in HPE. There is no clear correlation between MERSQI scores and the number or type of learning modes reported, as both high‐ and low‐MERSQI papers explored similar learning modes without a consistent pattern of emphasis. Publications in medical students were the highest (n = 13),^29^, ^30^, ^31^, ^32^, ^33^, ^34^, ^35^, ^36^, ^37^, ^38^, ^39^, ^40^, ^41^ nursing (n = 9),^24^, ^42^, ^43^, ^44^, ^45^, ^46^, ^47^, ^48^, ^49^ dentistry (n = 4),^50^, ^51^, ^52^, ^53^ pharmacy (n = 4),^54^, ^55^, ^56^, ^57^ veterinary (n = 1).^58^ One study spanned multiple health‐related fields (n = 1),^5^ and one study involved both medicine and pharmacy (n = 1).^59^ In terms of the countries where these studies were conducted, China (n = 6)^24^, ^31^, ^34^, ^37^, ^41^, ^48^ and The United States (n = 5)^5^, ^35^, ^40^, ^47^, ^54^ have the highest number of publications meeting the inclusion and exclusion criteria.

### 3.2 GenAI technology and Laurillard's six learning modes

**FIGURE 1:**
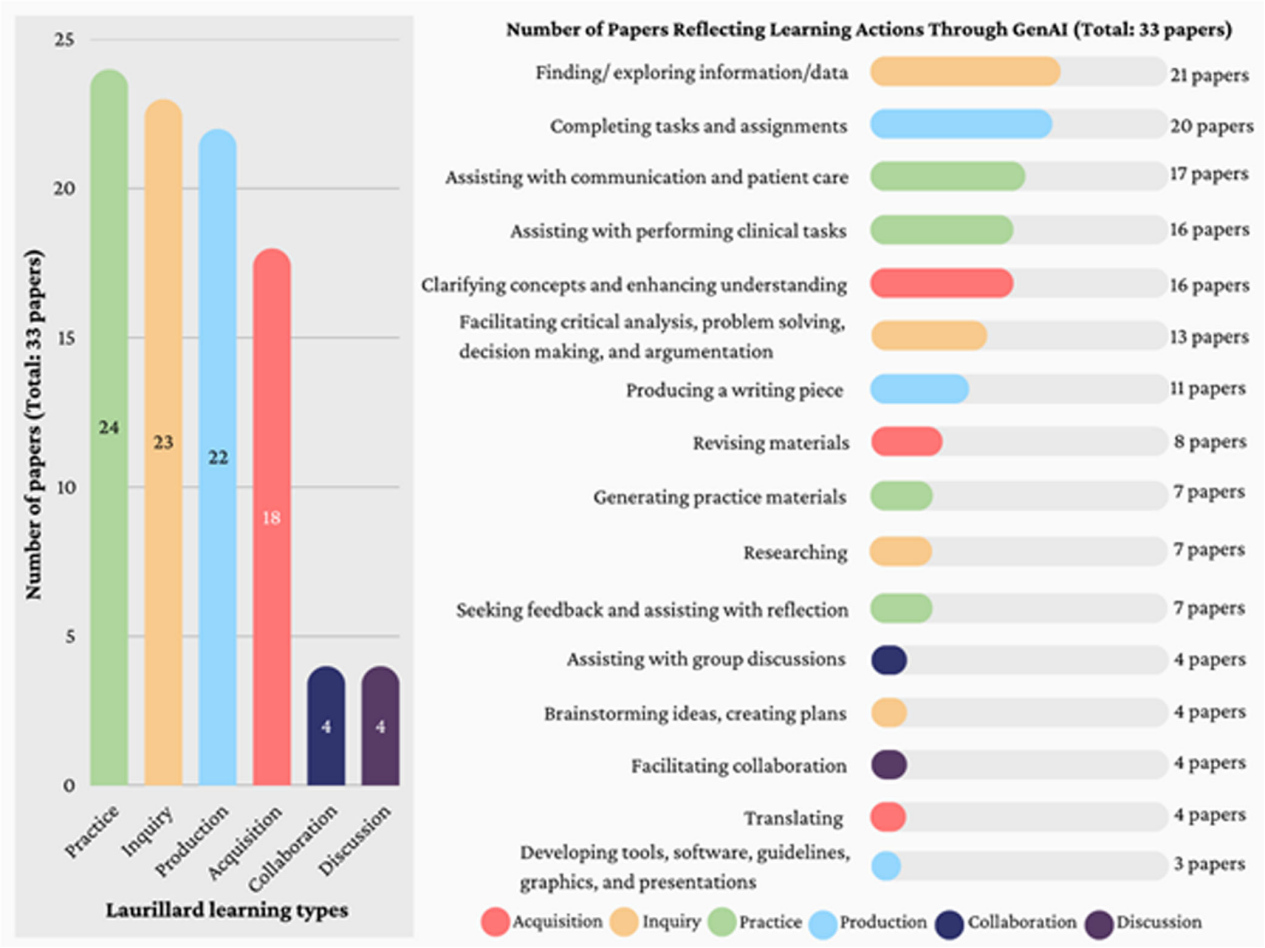
Laurillard learning types and GenAI uses. [Color figure can be viewed at wileyonlinelibrary.com]

Overall, GenAI has provided more advanced uses from convention and digital learning across *Practice*, *Acquisition*, *Inquiry* and *Production* such as deepening understanding, generating materials, supporting idea development and enabling practice in various forms. Notably, *Discussion* and *Collaboration* were less mentioned, suggesting a shift towards more individualised learning approaches in GenAI‐supported education. Out of all papers in the studied literature sample, *Practice* was the most frequently discussed mode, appearing in 24 papers (~73%). This was followed by Inquiry, covered in 23 papers (~70%) and Production, discussed in 22 papers (~67%). Acquisition appeared in 18 papers (~55%), whereas Discussion and Collaboration were each addressed in only 4 papers (~12%). Most of the included papers focused on ChatGPT, with a few mentioning other tools like custom GPT‐3.5, PowerPointAI, Termbot and other LLMs, highlighting the growing range of GenAI tools. The specific applications of GenAI described in each paper are listed in Appendix S5, and a detailed summary of how many papers addressed each learning mode is presented in Figure 1.

### 3.3 LEARNING MODE: ACQUISITION (n = 18)^24^, ^31^, ^33^, ^34^, ^35^, ^36^, ^37^, ^38^, ^42^, ^43^, ^44^, ^46^, ^49^, ^51^, ^52^, ^54^, ^57^, ^59^

Overall, 16 studies demonstrated GenAI's role in clarifying concepts, correcting misconceptions and simplifying complex topics to enhance learning.^33^, ^35^, ^36^, ^37^, ^38^, ^42^, ^43^, ^44^, ^46^, ^49^, ^51^, ^52^, ^54^, ^57^, ^59^ Students found GenAI faster and more accessible than traditional resources, as it allowed efficient, tailored access to information without needing multiple sources.^51^, ^57^ Additionally, students valued GenAI's mobile accessibility, using it to practice and learn medical terminology anytime.^38^

There were eight papers exploring GenAI's summarising ability, which helped students condense lengthy texts and lecture notes for efficient study preparation.^24^, ^31^, ^34^, ^35^, ^44^, ^51^, ^52^, ^54^ ChatGPT was especially effective for creating concise summaries of lecture outlines, enabling students to focus more time on other studies.^54^ Finally, four studies discussed GenAI's translation capabilities, helping students in multilingual settings reduce reliance on external tools and gain confidence with language support.^33^, ^35^, ^44^, ^54^

### 3.4 LEARNING MODE: INQUIRY (n = 23)^5^, ^24^, ^29^, ^31^, ^33^, ^34^, ^35^, ^36^, ^37^, ^38^, ^39^, ^40^, ^41^, ^43^, ^44^, ^45^, ^50^, ^51^, ^52^, ^54^, ^56^, ^57^, ^59^

There were 21 studies that discussed how GenAI supports efficient information acquisition by HPE students, enabling rapid, organised responses across queries.^5^, ^24^, ^31^, ^33^, ^34^, ^35^, ^36^, ^38^, ^39^, ^40^, ^41^, ^43^, ^44^, ^45^, ^50^, ^51^, ^52^, ^54^, ^56^, ^57^, ^59^ It was found that GenAI enables rapid, organised responses across diverse queries while minimising the need for checking multiple sources, increasing the speed of information gathering compared to traditional methods.^51^ However, while it provides seemingly accurate information, it sometimes lacks depth, currency or specific details, requiring verification against traditional sources.^51^, ^52^, ^57^ Of the 23 studies, 13 studies showed GenAI helps students think critically, solve problems, make decisions and explore different ideas by guiding them through complex scenarios and suggesting alternative solutions.^24^, ^29^, ^31^, ^33^, ^35^, ^36^, ^37^, ^39^, ^43^, ^45^, ^51^, ^52^, ^57^ However, concerns about information reliability highlighted the need for fact‐checking GenAI responses.^35^, ^57^

Seven studies highlighted GenAI's role in locating primary literature and citations, streamlining research.^5^, ^29^, ^31^, ^35^, ^41^, ^44^, ^54^ In the study by Ganjavi,^35^ most respondents noted that ChatGPT improved their research productivity and saved time on research tasks. Four studies showed GenAI's utility in brainstorming ideas, support ideation and project planning, helping students generate ideas and organise tasks.^5^, ^29^, ^35^, ^39^

### 3.5 LEARNING MODE: PRACTICE (n = 24)^5^, ^24^, ^29^, ^30^, ^31^, ^32^, ^33^, ^34^, ^35^, ^36^, ^37^, ^38^, ^39^, ^42^, ^43^, ^44^, ^49^, ^50^, ^51^, ^52^, ^54^, ^56^, ^57^, ^59^

There were 17 studies that described how HPE students can practise virtual patient interactions, perform history‐taking activities, enhance communication skills, improve interprofessional collaboration and promote effective patient interactions.^30^, ^31^, ^32^, ^33^, ^35^, ^36^, ^37^, ^42^, ^43^, ^44^, ^47^, ^49^, ^50^, ^51^, ^52^, ^54^, ^56^ In general, GenAI delivered accurate responses when following structured scripts and effectively simulated real patient scenarios; however, its response clarity and plausibility decreased with open‐ended or ambiguous questions, leading medical and nursing students to encounter inaccuracies or illogical answers in unscripted scenarios.^30^, ^42^ HPE students also used ChatGPT to enhance patient counselling and supported interprofessional communication across study fields.^47^, ^50^, ^56^ Additionally, 16 papers highlighted GenAI's role in clinical tasks by assisting students in creating treatment plans, managing medications, identifying drug interactions, explaining pathologies and supporting diagnostic skills, clinical reasoning, physical examination, history taking, drug monitoring and treatment planning through offering diagnostic prompts and aiding critical evaluation.^24^, ^29^, ^31^, ^33^, ^35^, ^36^, ^37^, ^39^, ^42^, ^43^, ^44^, ^51^, ^52^, ^54^, ^56^, ^57^ Reflective practice is another area where GenAI has shown to add value, with six studies noting its use for this purpose by students.^24^, ^38^, ^39^, ^43^, ^44^, ^56^, ^57^ However, students noted the absence of human interaction and detailed feedback, which raised concerns about GenAI's effectiveness in this category.^56^ In addition to reflection, GenAI also facilitates the generation of practice materials, as noted in seven papers.^5^, ^24^, ^29^, ^35^, ^52^, ^54^, ^59^ These studies highlight how HPE students used GenAI to generate diverse practice materials—including memory cards, clinical case scenarios, multiple‐choice questions and simulated exam scenarios through GenAI tools, enhancing interactive and tailored learning experiences.

### 3.6 LEARNING MODE: PRODUCTION (n = 22)^5^, ^24^, ^29^, ^33^, ^35^, ^36^, ^37^, ^40^, ^41^, ^43^, ^44^, ^46^, ^47^, ^48^, ^51^, ^52^, ^53^, ^54^, ^55^, ^57^, ^58^, ^59^

Twenty papers described GenAI assisting HPE students in completing tasks and assignments by simplifying complex information and reducing cognitive load for faster completion.^5^, ^24^, ^33^, ^35^, ^36^, ^37^, ^40^, ^41^, ^43^, ^44^, ^46^, ^48^, ^51^, ^52^, ^53^, ^54^, ^55^, ^57^, ^58^, ^59^ Moderate improvements in student grades and task completion times were noted when ChatGPT was utilised.^44^, ^48^, ^53^ In addition, there were 11 papers describing how GenAI platforms can assist in creating essays and case reports.^5^, ^29^, ^35^, ^41^, ^44^, ^48^, ^51^, ^52^, ^53^, ^54^, ^58^ ChatGPT effectively supported students in enhancing cohesion, argument clarity, organisation, language quality, logic, structure and grammar in their writing.^35^, ^43^, ^54^ However, concerns arose over potential over‐reliance, with occasional inaccuracies and misleading citations reducing its reliability for more complex academic work.^52^ Students had high expectations of ChatGPT's ability to improve their written products, but many found it more suitable for basic tasks like grammar correction and translation rather than complex academic writing.^53^ Furthermore, four studies highlighted GenAI's role in creating diverse educational materials and practical tools, with students generating visual aids, summaries, tables and clinical tools.^5^, ^35^, ^44^, ^52^

### 3.7 LEARNING MODE: DISCUSSION (n = 4)^24^, ^38^, ^43^, ^57^

The review identified that discussions involving GenAI were highlighted in four studies. Hamid et al^57^ noted that ChatGPT helped students voice opinions more freely by reducing fear of judgement, as they attributed errors to the tool and benefited from its quick responses for guidance in discussions. In other studies, students used GenAI to discuss health education projects and clinical cases, supporting guided discussions with peers and instructors.^24^, ^43^

### 3.8 LEARNING MODE: COLLABORATION (n = 4)^33^, ^38^, ^43^, ^57^

Four papers examined ChatGPT's role in collaboration, though details on how ChatGPT specifically facilitated teamwork were generally limited, highlighting the need for clearer guidance in collaborative settings. Hamid et al^57^ highlighted GenAI's positive impact on teamwork, though Shin et al^43^ Alnaim et al^33^ reported no significant effects on collaboration skills, with mixed views across education levels and a collaborative environment supporting self‐directed learning.

## 4 DISCUSSION

The higher percentage of papers discussing *Inquiry*, *Practice*, *Acquisition* and *Production* suggests that GenAI is being primarily used to support personalised, self‐directed learning, rather than promoting group interaction through *Discussion* and *Collaboration*, which were discussed far less frequently. Table 1 shows our proposed expansion to Laurillard^13^’s examples of how GenAI technologies may be applied within the learning activity types of the Conversational Framework.

**TABLE 1:** Adaptation of Laurillard's learning framework with examples of GenAI use.

Learning through	Conventional technology	Digital technology	Gen‐AI technology
Acquisition	Reading books, papers; Listening to teacher presentations face‐to‐face, lectures; Watching demonstrations, master classes	Reading multimedia, websites, digital documents and resources; Listening to podcasts, webcasts; Watching animations, videos	Reviewing, summarising and combining information from multiple resources, clarifying complex topics and translating information
Inquiry	Researching physical textbooks and journal articles in libraries	Accessing online medical journals, multimedia case studies, recorded lectures or podcasts on clinical topics.	Processing online data to answer a given question, analysing content relevance, gathering and showcasing available online data, exploring solutions and developing ideas for projects.
Practice	Clinical skills labs with hands‐on practice, manual simulations for patient care, or procedural practice with physical tools.	Using virtual patient simulators, digital databases for clinical research, interactive tools for diagnosing case scenarios.	Generating practice materials, allowing for varied scenarios of virtual patient simulators, delivering feedback on clinical tasks, and prompting reflective analysis.
Production	Producing written case studies, compiling portfolios with physical copies of assessments and diagnostic reports.	Creating e‐portfolios with digital documents of patient case studies, uploading diagnostic assessments and reflections.	Drafting essays, generating diagnostic summaries, creating reflective notes, producing visual aids and developing graphics or presentations for assignments.
Collaboration	Collaborative group work to create a joint output on paper e.g. co‐developing patient care plans on paper, sharing physical patient reports.	Collaborating on a joint output clinical case reports using shared online documents (Google Docs), engaging in breakout rooms for group discussions.	Collaborating with AI to brainstorm ideas (ideation), generate initial drafts for group projects
Discussion	Face‐to‐face group discussions in classrooms or clinics, in‐person debates or panel discussions regarding patient case studies.	Hosting virtual group discussions on clinical scenarios using Zoom or Canvas, interactive discussions through chat or forums.	Engaging with AI‐driven discussion prompts, using chatbots to simulate debates

With our proposal in Table 1, we provide educators a birds eye view of where to put their efforts and when GenAI can be an additional approach alongside conventional and digital technology due to its efficiency, wide reach and being readily accessible. ChatGPT and other GenAI tools have been shown to generate human‐like responses and mimic human interactions effectively.^30^, ^32^, ^50^, ^52^, ^55^ Its ability to provide instant, organised responses helps students bypass extensive searches, enabling faster learning tailored to student needs without relying on multiple sources.^51^, ^60^ Beyond these benefits, GenAI supports learning activities that are typically time‐consuming or difficult to accomplish without such technology, including the creation of practice materials and the provision of feedback.^61^, ^62^, ^63^, ^64^, ^65^ Menon and Shilpa's study,^66^ showed GenAI's ease of use and time‐saving features made it highly appealing to students, encouraging more frequent use by simplifying tasks and boosting productivity. Combined with its “anytime and anywhere” accessibility, GenAI provides students with valuable autonomy in their learning.^67^ This autonomy contributes to a more personalised learning experiences, which in turn boosts student engagement, motivation and proficiency by aligning educational content with individual interests, goals and knowledge levels.^68^, ^69^ However, this emphasis on personalised learning may reduce peer collaboration, as some students could rely on GenAI responses instead of engaging with classmates, potentially leading to decreased social interaction.^59^, ^66^, ^70^, ^71^ These factors raise the question of whether GenAI promotes individualised learning at the expense of human interaction, substituting other parties in collaboration and discussion learning mode.^72^ This lack of focus on social elements is also reflected in the literature, where fewer examples of discussion and collaboration are reported in the context of GenAI use in HPE.

This study underscores significant academic and research implications, highlighting both opportunities and challenges. A key implication is the need for more evidence on whether using GenAI, as opposed to conventional or digital technologies, impacts academic outcomes or clinical competency—an issue also highlighted in the systematic review by Feigerlova et al^10^ Ensuring the accuracy and reliability of GenAI outputs remains a major concern, given ongoing issues such as hallucinations, outdated information and a lack of source transparency.^50^, ^52^, ^58^ In HPE, where rigorous data quality is critical, students must be equipped to identify and address potential inaccuracies or biases in GenAI‐generated content.^30^, ^72^ GenAI integration also requires strategic planning and adherence to sound pedagogical principles.^73^ Additionally, overreliance on GenAI tools may hinder the development of critical thinking and independent learning skills, which are essential for professional competence.^32^, ^74^ To address these concerns, curriculum designers can consider implementing prompt engineering training programs, which could enhance students' critical thinking, improve GenAI response quality, reduce inaccuracies and align educational outcomes with personalised learning experiences.^75^ Institutions and educators must also update policies and strategies to uphold academic integrity, prevent plagiarism and ensure the ethical use of GenAI in HPE.^8^, ^75^, ^76^ Furthermore, the impact of GenAI on collaboration and discussion in healthcare education cannot be overlooked. Research highlights the importance of teamwork in healthcare, as its quality directly influences care delivery.^77^, ^78^ Future studies should explore how GenAI can enhance teamwork activities, particularly by improving the design of AI tools to better support social and emotional interactions. This could boost self‐efficacy in interprofessional communication and promote more effective collaboration.^10^

Like all systematic reviews, this review is subject to publication bias, particularly given the rapid release of GenAI studies. Limiting the search to English may exclude valuable international research; however, previous studies suggest minimal impact on overall conclusions.^79^, ^80^ Perceptions of GenAI users as less capable may influence self‐reported data and introduce bias in findings.^51^, ^65^ Additionally, known gender, racial and other biases in GenAI tools warrant further study into their effects on student learning.^81^, ^82^, ^83^ Despite these limitations, we followed a rigorous and transparent methodology. The protocol was registered with PROSPERO, ensuring transparency and efforts to reduce bias.^84^ We also adhered to PRISMA 2020 guidelines to support accurate and complete reporting.^85^ A comprehensive search across five databases and synthesis using Laurillard's learning modes provide a strong foundation for understanding GenAI use in HPE and guiding future work.

In conclusion, this paper proposes an expansion of Laurillard's framework based on current evidence of GenAI use in HPE to showcase how GenAI provides a different approach to learning from digital and conventional technologies. Further research is essential to fully understand the long‐term effects of GenAI on learners, educators and healthcare outcomes. As research continues, the potential for additional applications and learning types may emerge, further shaping the future of HPE.

## AUTHOR CONTRIBUTIONS

**Thai Pham:** Conceptualization (lead); methodology (lead); writing—original draft (lead); formal analysis (lead); writing—review and editing (equal). **Nilushi:** Methodology (equal); data collection (lead): formal analysis (lead); writing—review and editing (equal). **Betty:** Methodology (equal); formal analysis (lead); writing—review and editing (equal). **Danny:** Methodology (equal); formal analysis (lead); writing—review and editing (equal). **Travis:** Formal analysis (equal); writing—review and editing (equal). **Elizabeth:** Conceptualization (equal); writing—original draft (equal); writing—review and editing (equal) **Angelina Lim:** Conceptualization (lead); methodology (lead); writing—original draft (lead); formal analysis (lead); writing—original draft (lead); writing—review and editing (equal).

## CONFLICT OF INTEREST STATEMENT

The authors declare no conflicts of interest.

## ETHICS STATEMENT

This review did not require any ethics approval as no human data was collected.

## Supporting information


**Appendix S1.** Systematic search terms across all five databases.

**Appendix S2.** Eligibility criteria.

**Appendix S3.** Laurillard modes and GenAI uses: Definitions of learning actions.

**Appendix S4.** PRISMA Flowchart showcasing screening process.

**Appendix S5.** A detailed Summary of included articles (n = 33).

## Data Availability

The data that support the findings of this study are available from the corresponding author upon reasonable request.
