# Supplementary material for: The impact of generative AI on health professional education: A systematic review in the context of student learning
Source: Med Educ. 2025 Jun 18;59(12):1280–9. doi: 10.1111/medu.15746 (PMC12686775; doi:10.1111/medu.15746)
Supplement: Supplementary file 1 — Appendix S1. Systematic search terms across all five databases. [file MEDU-59-1280-s001.docx]

**Appendix S1. Systematic search terms across all five databases**

|  | **Concept 1 (GenAI)** | **Concept 2 (Health Education)** |
| --- | --- | --- |
| **Embase and MEDLINE** | - Generative artificial intelligence  - Generative AI  - GenAI  - Natural Language Processing  - ChatGPT  - GPT  - Chatbot  - Bard  - Copilot  - Gemini  - Large language model  - Large language models | - (Dent* adj3 education)  - (Chiro* adj3 education)  - (Chinese medic* adj3 education)  - (Medic* adj3 education)  - (Nurs* adj3 education)  - (Optom* adj3 education)  - (Osteop* adj3 education)  - (Pharm* adj3 education)  - (Physiot* adj3 education)  - (Podiat* adj3 education)  - (Psychol* adj3 education)  - (Health profession* adj3 education)  - (Midw* adj3 education)  - (("Torres Strait" or "Aborigin*" or Indigenous) and "Health" and "Education")  - (Medical radia* adj3 education)  - (Occupational thera* adj3 education)  - (Paramed* adj3 education)  - education, continuing/ or education, dental, continuing/ or education, medical, continuing/ or education, nursing, continuing/ or education, pharmacy, continuing/ or education, professional, retraining/ or education, dental/ or education, dental, graduate/ or education, graduate/ or "education, biological and biomedical sciences, graduate"/ or education, medical, graduate/ or education, nursing, graduate/ or education, pharmacy, graduate/ or education, medical/ or education, medical, undergraduate/ or education, nursing/ or education, nursing, associate/ or education, nursing, baccalaureate/ or education, nursing, diploma programs/ or nursing education research/ or education, pharmacy/ or pharmacy residencies/ or education, public health professional/ or education, veterinary |
| **Scopus** | ( TITLE-ABS-KEY ( "generative artificial intelligence" ) OR TITLE-ABS-KEY ( "generative ai" ) OR TITLE-ABS-KEY ( "genai" ) OR TITLE-ABS-KEY ( "natural language processing" ) OR TITLE-ABS-KEY ( "chatgpt" ) OR TITLE-ABS-KEY ( "gpt" ) OR TITLE-ABS-KEY ( "chatbot" ) OR TITLE-ABS-KEY ( "bard" ) OR TITLE-ABS-KEY ( "copilot" ) OR TITLE-ABS-KEY ( "gemini" ) ) | ( TITLE-ABS-KEY ( education W/3 ( ( dent* ) OR ( chiro* ) OR ( "chinese medicine" ) OR ( medic* ) OR ( nurs* ) OR ( opto* ) OR ( osteop* ) OR ( pharm* ) OR ( physiot* ) OR ( podiat* ) OR ( psychol* ) OR ( "health profession*" ) OR ( midw* ) OR ( ( "torres strait" OR "aborigin*" OR indigenous ) AND ( "health" ) ) OR ( "medical radia*" ) OR ( "occupational thera*" ) ) ) ) |
| **Education Database & ERIC** | (noft(("generative artificial intelligence") OR ("generative ai") OR ("genai") OR ("natural language processing") OR ("chatgpt") OR ("gpt") OR ("chatbot") OR ("bard") OR ("copilot") OR ("gemini")) | (education AND ((dent*) OR (chiro*) OR ("chinese med*") OR (medic*) OR (nurs*) OR (opto*) OR (osteop*) OR (pharm*) OR (physiot*) OR (podiat*) OR (psychol*) OR ("health profession*") OR (midw*) OR (("Torres Strait" OR "Aborigin*" OR indigenous) AND ("Health")) OR ("medical radia*") OR ("Occupational thera*") OR ("paramed*")))) AND (at.exact("Article") AND stype.exact("Scholarly Journals")) |
