# Supplementary material for: The impact of generative AI on health professional education: A systematic review in the context of student learning
Source: Med Educ. 2025 Jun 18;59(12):1280–9. doi: 10.1111/medu.15746 (PMC12686775; doi:10.1111/medu.15746)
Supplement: Supplementary file 2 — Appendix S2. Eligibility criteria. [file MEDU-59-1280-s003.docx]

#### **Appendix S2. Eligibility criteria**

| **Criterion** |  | **Inclusion/Exclusion** | **Description** | **Justification** |
| --- | --- | --- | --- | --- |
| Study design |  | Inclusion | Peer-reviewed empirical studies with original research findings. | Ensures studies are based on robust evidence and present new research data. |
|  |  | Exclusion | Editorials, commentaries, opinions, and reviews. | The intent of this review is to capture primary literature and avoid duplication. |
| Population |  | Inclusion | Studies involving health profession students (list adapted from WHO health workers) in higher education.   - Medical Practitioner/Doctor - Nurse/Nurse Practitioner - Pharmacist - Dentist - Physiotherapist - Psychologist - Midwife - Occupational therapist - Paramedic - Optometrist - Chiropractor - Chinese Medical Practitioner - Osteopath - Medical Radiation specialist - Indigenous health practitioner - Podiatrist - Vet | Focus is on health profession education, ensuring the target audience is clearly defined. |
|  |  | Exclusion | Studies in non-tertiary settings | Maintains the relevance and specificity of the review to Health Professional Education students to inform guidance for academics in higher education |
| Intervention |  | Inclusion | Studies were included if they involved students directly using GenAI like GPT-3 or GPT-4 to support their learning activities. This includes students' active engagement with GenAI for tasks such as research, problem-solving, or content creation within formal educational settings. | The review aims to assess the direct impact of GenAI on student learning, focusing on how these tools enhance educational experiences and promote active student involvement, rather than passive use or non-educational applications. |
|  |  | Exclusion | Studies without GenAI components or direct student use of GenAI for learning or where GenAI was used for personal, non-educational purposes. | This review specifically focuses on the unique capacities of GenAI. |
| Outcome |  | Inclusion | Studies investigating student’s use of GenAI | The review focuses on how GenAI is being used by students |
|  |  | Exclusion | Studies without any data related to actual student use or uses unrelated to education | Irrelevant outcomes do not meet the core interest of the review |
| Language |  | Inclusion | Articles published in English. | Ensures language consistency and ease of interpretation for the review team. |
|  |  | Exclusion | Non-English publications. | Whilst this may be considered a limitation, we found some of the Non-English studies were hard to interpret and decided to exclude these to ensure the team was presenting accurately interpreted data. |
