# Supplementary material for: The impact of generative AI on health professional education: A systematic review in the context of student learning
Source: Med Educ. 2025 Jun 18;59(12):1280–9. doi: 10.1111/medu.15746 (PMC12686775; doi:10.1111/medu.15746)
Supplement: Supplementary file 3 — Appendix S3. Laurillard modes and GenAI uses: Definitions of learning actions. [file MEDU-59-1280-s005.docx]

#### **Appendix S3. Laurillard modes and GenAI uses: Definitions of learning actions**

| **Laurillard modes** | **GenAI uses** | **Definitions** |
| --- | --- | --- |
| Acquisition | Clarifying concepts and enhancing understanding | To clarify concepts and enhance understanding, provide insights, address misconceptions, and simplify complex topics, helping students resolve doubts and improve knowledge retention |
|  | Revising materials | To assist students in summarizing and reviewing information by condensing lengthy texts, lecture outlines, and concepts into concise, understandable overviews. This process enhances comprehension, organizes information, and supports task preparation, enabling more efficient learning, study sessions, and project completion. |
|  | Translating | To assist with translation by helping students convert information into different languages, overcoming language barriers, and enhancing learning for those with English as a second language, improving efficiency and comprehension in multilingual contexts. |
| Collaboration | Facilitating collaboration | To assist students in working together, sharing knowledge, and contributing towards common goals by fostering teamwork and collaborative efforts. |
| Discussion | Assisting with group discussions | To assist with group discussions by stimulating meaningful conversations |
| Inquiry | Brainstorming, creating plans | To generate and organise diverse ideas, often with the assistance of tools like ChatGPT, which supports the development of project frameworks, research topics, and structured plans by providing examples and enhancing creative thinking. |
|  | Facilitating Critical Analysis, Problem-Solving, Decision-Making, and Refining Arguments | To assist students in analysing and evaluating information, helping them critically assess the credibility and relevance of content, and/or guiding them through solving problems and generating solutions in complex scenarios. This involves enhancing their critical thinking and decision-making abilities, particularly in clinical reasoning, by encouraging the exploration of multiple solutions and perspectives, ultimately enabling them to build arguments. |
|  | Finding/ exploring information/ data | To assist students in acquiring information and data by quickly providing relevant resources, answering specific content questions, and offering supplemental knowledge, making the information-gathering process more efficient for academic tasks. |
|  | Researching | To assist with research by helping students quickly look up information, such as finding studies, primary literature, and citations, streamlining the research process and improving efficiency in gathering relevant academic resources. |
| Practice | Assisting with communication and patient care | To gather a patient's medical and social information through structured questioning, and with the support of GenAI, students can practise virtual patient interactions, refining their questioning techniques. Additionally, GenAI assists in conveying information and improving healthcare by enhancing interprofessional collaboration, counselling, patient interactions, and professional conduct through improved communication skills and educational resources. |
|  | Assisting with performing clinical tasks | Assisting with clinical tasks involves developing essential skills such as conducting physical examinations, creating treatment plans and patient notes, managing medications, and identifying and resolving issues like drug interactions and adverse reactions. This also includes making informed treatment decisions, explaining pathologies, and predicting patient outcomes to provide comprehensive care. Additionally, learning to use systems efficiently requires understanding how to navigate and utilise various tools or platforms effectively, enabling better management of clinical tasks and ensuring optimal use of resources. |
|  | Generating practice materials & Producing memory cards | To create study resources such as quizzes, case scenarios, and learning guides, improving exam preparation and comprehension and/or to generate flashcards, such as Anki© cards, and clarify difficult concepts, tools like ChatGPT help students study efficiently by organizing information into easy-to-review formats for better retention and understanding. |
|  | Seeking feedback and Assisting with reflection | To support self-assessment by encouraging students to evaluate their learning, identify areas for growth, and create improvement plans based on critical reflection and feedback, while also assisting them in seeking feedback by providing immediate responses and corrections to help validate their understanding. |
| Production | Completing tasks and assignments | To complete tasks or assignments involves efficiently organising tasks, and managing assignments, ensuring timely completion of academic responsibilities while maintaining quality and accuracy throughout the process. |
|  | Developing tools, software, guidelines, graphics, and presentations | To assist students in developing tools, software, and presentations by providing GenAI-generated outputs that align with real-world healthcare applications. This includes creating patient teaching tools, care guidelines, policy frameworks, and structured content such as PowerPoint templates, summaries, and visual aids like images and charts, while also offering alternative approaches to information presentation. |
|  | Producing a written piece | To draft and/or produce essays and case reports, assist with creating outlines, proofreading, enhancing idea cohesion, and paraphrasing or rephrasing content to improve clarity and structure in reflective pieces and academic writing. |
