# Supplementary figures and images for: The impact of generative AI on health professional education: A systematic review in the context of student learning
Source: Med Educ. 2025 Jun 18;59(12):1280–9. doi: 10.1111/medu.15746 (PMC12686775; doi:10.1111/medu.15746)

**Appendix S4. PRISMA Flowchart showcasing screening process**
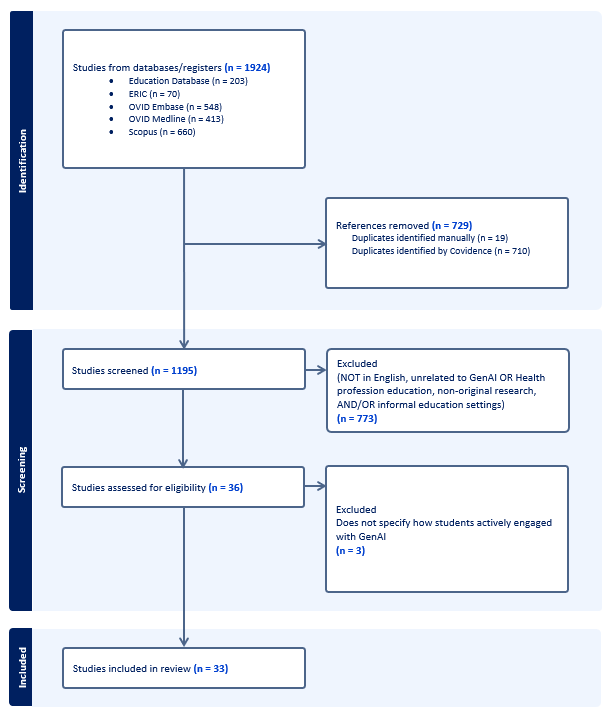

Supplement: Supplementary file 4 — Appendix S4. PRISMA Flowchart showcasing screening process. [file MEDU-59-1280-s004.docx]
