# Supplementary material for: The impact of generative AI on health professional education: A systematic review in the context of student learning
Source: Med Educ. 2025 Jun 18;59(12):1280–9. doi: 10.1111/medu.15746 (PMC12686775; doi:10.1111/medu.15746)
Supplement: Supplementary file 5 — Appendix S5. A detailed Summary of included articles (n = 33). [file MEDU-59-1280-s002.docx]

**Appendix S5. A detailed Summary of included articles (n=33)**

| **Author(s) and year** | **Title** | **Country** | **Field(s)** | **Study Design** | **GenAI platform(s)** | **Data Collection methods** | **Aims/Objectives** | **Main Results** | **Laurillard Learning Type** | **Specific learning mentioned** | **MERSQI score** |
| --- | --- | --- | --- | --- | --- | --- | --- | --- | --- | --- | --- |
| Alkhaaldi et al., 2023^30^ | Medical Student Experiences and Perceptions of ChatGPT and Artificial Intelligence: Cross-Sectional Study. | United Arab Emirates | Medicine | Retrospective Survey/Interview on a Previously Administered Intervention | ChatGPT | Cross-sectional survey | To explore medical students' experiences and perceptions of ChatGPT and AI in their medical training and future careers. | Most students had limited experience with ChatGPT during medical school but expressed optimism about its future role in medical education and healthcare | Inquiry, Practice, Production | Facilitating Critical Analysis, Problem-Solving, Decision-Making, and Refining Arguments; Researching; Brainstorming, creating plans; Assisting with performing clinical tasks; Generating practice materials & Producing memory cards; Producing a writing piece | 9 |
| Almazrou et al., 2024^40^ | Enhancing medical students critical thinking skills through ChatGPT: An empirical study with medical students | Saudi Arabia | Medicine | Retrospective Survey/Interview on a Previously Administered Intervention | ChatGPT | Cross-sectional survey | To assess the effectiveness of ChatGPT in enhancing critical thinking skills among medical students. | ChatGPT was found to improve critical thinking skills, particularly in encouraging questioning, fostering problem-solving, and simulating real-world scenarios​ | Inquiry, Practice | Finding/ exploring information/ data; Facilitating Critical Analysis, Problem-Solving, Decision-Making, and Refining Arguments; Brainstorming, creating plans; Assisting with performing clinical tasks; Seeking feedback and assisting with reflection | 12.5 |
| Alnaim et al., 2024^34^ | Effectiveness of ChatGPT in remote learning environments: An empirical study with medical students in Saudi Arabia | Saudi Arabia | Medicine | Retrospective Survey/Interview on a Previously Administered Intervention | ChatGPT | Cross-sectional survey | To assess the effectiveness of ChatGPT in remote learning environments for medical students | AI tools like ChatGPT enhanced learning outcomes, with students appreciating the usefulness of these tools in their learning process​ | Inquiry, Practice, Production, Acquisition, Collaboration | Finding/ exploring information/ data; Facilitating Critical Analysis, Problem-Solving, Decision-Making, and Refining Arguments; Assisting with communication and patient care; Assisting with performing clinical tasks; Completing tasks and assignments; Clarifying concepts and enhancing understanding; Translating; Facilitating collaboration | 11 |
| Anderson et al., 2024^56^ | Pharmacy student use of ChatGPT: A survey of students at a U.S. School of Pharmacy | United States | Pharmacy | Retrospective Survey/Interview on a Previously Administered Intervention | ChatGPT | Cross-sectional survey | To investigate how PharmD students use ChatGPT for personal, academic, and clinical reasons, and whether they think ChatGPT training should be part of their curriculum. | Nearly half of the students used ChatGPT, mainly for personal and academic purposes, and many expressed interest in learning how to use it effectively in their education | Inquiry, Practice, Production, Acquisition | Finding/ exploring information/ data; Researching; Assisting with communication and patient care; Assisting with performing clinical tasks; Generating practice materials and producing memory cards; Completing tasks and assignments; Producing a writing piece; Clarifying concepts and enhancing understanding; Revising materials; Translating | 6 |
| Araji and Brooks, 2024^41^ | Evaluating The Role of ChatGPT as a Study Aid in Medical Education in Surgery. | United States | Medicine | Empirical Research with an Active Intervention | ChatGPT | Randomised crossover study | To compare the effectiveness of ChatGPT and Google search as study aids during a surgery rotation and evaluate students’ willingness to use ChatGPT | ChatGPT improved students' post-assessment scores, but most were still reluctant to use it during their surgery clerkship | Inquiry, Production | Finding/ exploring information/ data; Completing tasks and assignments | 12.5 |
| Ba, Zhang and Yi, 2024^38^ | Enhancing clinical skills in pediatric trainees: a comparative study of ChatGPT-assisted and traditional teaching methods | China | Medicine | Both | ChatGPT | Randomised controlled experimental design | To compare the effectiveness of ChatGPT-assisted instruction and traditional teaching methods on pediatric trainees' clinical skills. | ChatGPT-assisted students showed higher improvement in theoretical aspects but not in hands-on skills. | Inquiry, Practice, Production, Acquisition | Facilitating Critical Analysis, Problem-Solving, Decision-Making, and Refining Arguments; Assisting with communication and patient care; Assisting with performing clinical tasks; Completing tasks and assignments; Clarifying concepts and enhancing understanding | 12.5 |
| Bentafah et al., 2024^43^ | Assessing the efficacy of ChatGPT as a virtual patient in nursing simulation training: A study on nursing students' experience | Morocco | Nursing | Both | ChatGPT | Mixed methods (Qualitative Analysis followed by Likert scale surveys and performance evaluations) | To assess the effectiveness of ChatGPT as a virtual patient in nursing simulation training | Students found ChatGPT effective in improving clinical communication and interaction skills during virtual patient simulations​ | Practice, Acquisition | Assisting with communication and patient care; Assisting with performing clinical tasks; Clarifying concepts and enhancing understanding | 10.5 |
| Bumbach et al., 2024^49^ | The use of artificial intelligence for graduate nursing education: An educational evaluation | United States | Nursing | Retrospective Survey/Interview on a Previously Administered Intervention | ChatGPT | Cross-sectional survey | To evaluate the use of AI in graduate-level nursing education | ChatGPT was effective for theory-based tasks, but students had challenges in applying it to complex clinical scenarios. | Practice, Production | Assisting with communication and patient care; Developing tools/software and guidelines | 8 |
| Chang et al., 2023^47^ | Facilitating nursing and health education by incorporating ChatGPT into learning designs | Taiwan | Nursing | Both | ChatGPT | Mixed methods (Comparison of student performance through exams and surveys) | To explore how ChatGPT can be integrated into learning designs for nursing and health education to enhance engagement and learning outcomes. | ChatGPT enhanced student engagement and learning, particularly in understanding complex topics, though concerns about its limitations and ethical considerations were raised​ | Inquiry, Practice, Production, Acquisition, Discussion | Finding/ exploring information/ data; Facilitating Critical Analysis, Problem-Solving, Decision-Making, and Refining Arguments; Assisting with performing clinical tasks; Seeking feedback and assisting with reflection; Generating practice materials and producing memory cards; Completing tasks and assignments; Clarifying concepts and enhancing understanding; Revising materials; Assisting with group discussions | 13.5 |
| Gan et al., 2024^35^ | Integrating ChatGPT in Orthopedic Education for Medical Undergraduates: Randomized Controlled Trial | China | Medicine | Empirical Research with an Active Intervention | ChatGPT | Randomised controlled trial | To assess ChatGPT’s accuracy in answering orthopaedics-related MCQs and evaluate its short- and long-term effectiveness as a learning aid. *** data regarding ChatGPT's accuracy in answering questions are not included in our analysis | ChatGPT improved students’ performance on orthopaedics-related MCQs and their final exams in other medical subjects​ | Inquiry, Acquisition | Finding/ exploring information/ data; Revising materials | 13.5 |
| Ganjavi et al., 2024^36^ | ChatGPT and large language models (LLMs) awareness and use. A prospective cross-sectional survey of U.S. medical students | United States | Medicine | Retrospective Survey/Interview on a Previously Administered Intervention | ChatGPT and other large language models (LLMs) | Prospective cross-sectional survey | To investigate the awareness and use of ChatGPT and LLMs among medical students | ChatGPT was commonly used for studying medical concepts, assisting with diagnosis/treatment plans, and academic writing tasks​ | Inquiry, Practice, Production, Acquisition | Finding/ exploring information/ data; Facilitating Critical Analysis, Problem-Solving, Decision-Making, and Refining Arguments; Researching; Brainstorming, creating plans; Assisting with communication and patient care; Assisting with performing clinical tasks; Generating practice materials and producing memory cards; Completing tasks and assignments; Producing a writing piece; Creating visual aids and materials; Developing tools/software and guidelines; Clarifying concepts and enhancing understanding; Revising materials; Translating | 9.5 |
| Hamid et al., 2023^59^ | Exploratory study on student perception on the use of chat AI in process-driven problem-based learning | Malaysia | Pharmacy | Both | ChatGPT | Qualitative exploratory case study | To explore pharmacy students’ perceptions of using ChatGPT in PDPBL sessions and its impact on their engagement and learning experience. | Students found ChatGPT enhanced engagement, collaboration, and learning, though concerns about the reliability of information were raised | Inquiry, Practice, Production, Acquisition, Collaboration, Discussion | Finding/ exploring information/ data; Facilitating Critical Analysis, Problem-Solving, Decision-Making, and Refining Arguments; Assisting with performing clinical tasks; Seeking feedback and assisting with reflection; Completing tasks and assignments; Clarifying concepts and enhancing understanding; Facilitating collaboration; Assisting with group discussions | 9 |
| Holderried et al., 2024^31^ | A Generative Pretrained Transformer (GPT)–Powered Chatbot as a Simulated Patient to Practice History Taking: Prospective, Mixed Methods Study | Germany | Medicine | Both | GPT3.5 - chatbot | Mixed methods study (Conversations between medical students and ChatGPT recorded and analysed, followed by the Chatbot Usability Questionnaire (CUQ).) | To explore the feasibility of using ChatGPT as a simulated patient to practice history taking in medical education. | ChatGPT provided mostly plausible responses, and students rated the experience as positive with a high usability score​ | Practice | Assisting with communication and patient care | 10 |
| Hsu, 2023^48^ | Mastering Medical Terminology with ChatGPT and Termbot | Taiwan | Nursing | Empirical Research with an Active Intervention | ChatGPT, Termbot | Pre-test and post-test evaluations on medical terminology knowledge | To evaluate the effectiveness of ChatGPT in teaching medical terminology | Both ChatGPT and Termbot improved students’ learning outcomes, with ChatGPT showing slightly better progress | Production, Acquisition | Completing tasks and assignments; Clarifying concepts and enhancing understanding | 12.5 |
| Huang & Lin, 2024^39^ | ChatGPT as a life coach for professional identity formation in medical education: A self-regulated learning perspective | Taiwan | Medicine | Empirical Research with an Active Intervention | ChatGPT | Qualitative descriptive study using the phenomenological method | To explore the impact of ChatGPT as a life coach on medical students’ professional identity formation through self-regulated learning. | ChatGPT increased motivation for planning professional identity formation, strengthened the mastery of professional identity, and broadened perspectives on professional identity formation. | Inquiry, Practice, Acquisition, Collaboration, Discussion | Finding/ exploring information/ data; Seeking feedback and assisting with reflection; Clarifying concepts and enhancing understanding; Facilitating collaboration; Assisting with group discussions | 8 |
| Jallad et al., 2024^51^ | Artificial intelligence tools utilized in nursing education: Incidence and associated factors | Palestine | Nursing | Retrospective Survey/Interview on a Previously Administered Intervention | ChatGPT, PowerPointAI data about mobile application excluded | Cross-sectional survey | To determine factors affecting the usefulness and sustainability of artificial intelligence tools in nursing education | AI tools like ChatGPT were effective in enhancing learning outcomes, improving satisfaction, and supporting the educational process for nursing students​ | Practice, Acquisition | Assisting with communication and patient care; Clarifying concepts and enhancing understanding | 8.5 |
| Kavadella et al., 2024^54^ | Evaluation of ChatGPT's Real-Life Implementation in Undergraduate Dental Education: Mixed Methods Study. | Cyprus | Dental | Both | ChatGPT | Mixed methods (Comparison of student performance through exams and thematic analysis of qualitative feedback via questionnaires) | To evaluate the real-life implementation of ChatGPT in undergraduate dental education and its impact on learning outcomes. | Students using ChatGPT performed better on exams compared to traditional literature-based groups and reported positive experiences with its use, despite some limitations like inaccurate citations | Inquiry, Practice, Production, Acquisition | Finding/ exploring information/ data; Facilitating Critical Analysis, Problem-Solving, Decision-Making, and Refining Arguments; Assisting with communication and patient care; Assisting with performing clinical tasks; Generating practice materials and producing memory cards; Completing tasks and assignments; Producing a writing piece; Creating visual aids and materials; Developing tools/software and guidelines; Clarifying concepts and enhancing understanding; Revising materials | 12.5 |
| Kazley et al., 2024^61^ | Is use of ChatGPT cheating? Students of health professions perceptions | United States | Multiple fields | Retrospective Survey/Interview on a Previously Administered Intervention | ChatGPT | Cross-sectional survey | To explore health professions students' perceptions of generative AI use and whether it constitutes cheating. | Students viewed using ChatGPT for exams or writing papers as cheating, but many considered its use for studying or researching acceptable​ | Inquiry, Practice, Production | Finding/ exploring information/ data; Researching; Brainstorming, creating plans; Generating practice materials and producing memory cards; Completing tasks and assignments; Producing a writing piece; Creating visual aids and materials | 9 |
| Li et al., 2024^42^ | Exploring the potential of artificial intelligence to enhance the writing of english academic papers by non-native english-speaking medical students - the educational application of ChatGPT | China | Medicine | Empirical Research with an Active Intervention | ChatGPT (specifically ChatGPT-3.5 and ChatGPT-4). | Experimental study with pre- and post-revision comparison. | To evaluate the effectiveness of ChatGPT in improving the English academic writing of non-native English-speaking medical students. | ChatGPT significantly improved students’ writing quality in terms of structure, logic, and language | Inquiry, Production | Finding/ exploring information/ data; Researching; Completing tasks and assignments; Producing a writing piece | 12 |
| Liu et al., 2024^50^ | Perception of ChatGPT by Nursing Undergraduates | China | Nursing | Both | ChatGPT | Mixed methods performance evaluation study (Performance metrics, user satisfaction surveys, and interviews with participants using ChatGPT for clinical reasoning) | To explore the impact of integrating ChatGPT into healthcare education to enhance student engagement and learning outcomes. | Students found ChatGPT useful in understanding complex healthcare topics, improving learning engagement, though concerns about AI reliability and ethics were raised​ | Production | Completing tasks and assignments; Producing a writing piece | 9 |
| Luo et al., 2023^46^ | College Students’ Employability, Cognition, and Demands for ChatGPT in the AI Era Among Chinese Nursing Students: Web-Based Survey | China | Nursing | Retrospective Survey/Interview on a Previously Administered Intervention | ChatGPT | Cross-sectional survey | To investigate Chinese nursing students’ artificial intelligence quotient (AIQ), employability, and demand for ChatGPT to guide future nursing education. | A majority of students had not used ChatGPT, and their AIQ was at an intermediate level, suggesting a need for increased AI literacy in nursing education | Inquiry | Finding/ exploring information/ data; Facilitating Critical Analysis, Problem-Solving, Decision-Making, and Refining Arguments | 11 |
| Mosleh et al., 2023^62^ | Medicine and Pharmacy Students’ Knowledge, Attitudes, and Practice regarding Artificial Intelligence Programs: Jordan and West Bank of Palestine | Jordan, West Bank of Palestine | Medicine and Pharmacy | Retrospective Survey/Interview on a Previously Administered Intervention | ChatGPT | Cross-sectional survey | To assess the knowledge, attitudes, and practices regarding AI programs, including ChatGPT, among medicine and pharmacy students in Jordan and the West Bank of Palestine. | While many students had heard of AI programs, fewer had used them in their studies, with significant differences between medicine and pharmacy students in terms of AI application for academic tasks​ | Inquiry, Practice, Production, Acquisition | Finding/ exploring information/ data; Generating practice materials and producing memory cards; Completing tasks and assignments; Clarifying concepts and enhancing understanding | 11 |
| Pereira et al., 2023^33^ | Designing and building OSCEBot for virtual OSCE - Performance evaluation | Portugal | Medicine | Empirical Research with an Active Intervention | OSCEBot, developed using ChatGPT and the SentenceTransformers (SBERT) framework. | Experimental performance evaluation | To develop and evaluate OSCEBot, a chatbot for training medical students in clinical interviews as part of their OSCE preparation. | OSCEBot improved clinical interviewing skills, with performance improving as cases were refined based on student feedback​ | Practice | Assisting with communication and patient care | 8 |
| Roganović, 2024^55^ | Familiarity with ChatGPT Features Modifies Expectations and Learning Outcomes of Dental Students | Serbia | Dental | Retrospective Survey/Interview on a Previously Administered Intervention | ChatGPT | Comparative experimental study - Surveys | To examine how familiarity with ChatGPT’s features affects dental students' expectations and learning outcomes in pharmacology | Familiarity with ChatGPT’s features improved quiz performance, even among students who did not use the tool, indicating an influence on expectations and learning outcomes​ | Production | Completing tasks and assignments; Producing a writing piece | 12.5 |
| Saha et al., 2024^52^ | Assessing the Awareness of Dental Undergraduates Regarding the Use of ChatGPT in Dentistry: A Questionnaire Survey | India | Dental | Retrospective Survey/Interview on a Previously Administered Intervention | ChatGPT | Cross-sectional survey | To evaluate the awareness and engagement of dental undergraduate students with ChatGPT in academic and clinical contexts | Most students were aware of ChatGPT and had used it for academic purposes, with a minority expressing concerns about its ethical implications | Inquiry, Practice | Finding/ exploring information/ data; Assisting with communication and patient care | 10 |
| Saravia‐Rojas et al., 2024^53^ | Artificial intelligence: ChatGPT as a disruptive didactic strategy in dental education | Peru | Dental | Retrospective Survey/Interview on a Previously Administered Intervention | ChatGPT | Comparative experimental study - Surveys | To evaluate the influence of ChatGPT on academic tasks performed by undergraduate dental students and assess students’ perceptions of its usefulness. | While ChatGPT was perceived as useful by students, traditional methods of searching scientific articles yielded higher scores in academic tasks | Inquiry, Practice, Production, Acquisition | Finding/ exploring information/ data; Facilitating Critical Analysis, Problem-Solving, Decision-Making, and Refining Arguments; Assisting with communication and patient care; Assisting with performing clinical tasks; Completing tasks and assignments; Producing a writing piece; Clarifying concepts and enhancing understanding; Revising materials | 7.5 |
| Shin et al., 2024^44^ | The Impact of Artificial Intelligence-Assisted Learning on Nursing Students' Ethical Decision-making and Clinical Reasoning in Pediatric Care A Quasi-Experimental Study | Republic of Korea | Nursing | Retrospective Survey/Interview on a Previously Administered Intervention | ChatGPT | Quasi-experimental study | To evaluate the impact of AI-assisted learning on nursing students' ethical decision-making and clinical reasoning in paediatric care | The control group outperformed the ChatGPT group in ethical standards and clinical reasoning, though ChatGPT improved time efficiency and offered diverse perspectives | Inquiry, Practice, Production, Acquisition, Collaboration, Discussion | Finding/ exploring information/ data; Facilitating Critical Analysis, Problem-Solving, Decision-Making, and Refining Arguments; Assisting with communication and patient care; Assisting with performing clinical tasks; Seeking feedback and assisting with reflection; Completing tasks and assignments; Clarifying concepts and enhancing understanding; Facilitating collaboration; Assisting with group discussions | 12.5 |
| Summers et al., 2024^45^ | Navigating challenges and opportunities: Nursing student's views on generative AI in higher education | Australia | Nursing | Retrospective Survey/Interview on a Previously Administered Intervention | ChatGPT and other generative AI tools | Qualitative descriptive study - Semi-structured interviews with nursing students | To explore nursing students' perspectives on the integration of generative AI tools into their studies. | Students expressed concerns about generative AI's impact on learning critical thinking skills and ethical considerations, but also acknowledged its potential to improve efficiency and productivity​ | Inquiry, Practice, Production, Acquisition | Finding/ exploring information/data; Researching; Assisting with communication and patient care; Assisting with performing clinical tasks; Seeking feedback and assisting with reflection; Completing tasks and assignments; Producing a writing piece; Creating visual aids and materials; Clarifying concepts and enhancing understanding; Revising materials; Translating | 8 |
| Svendsen et al., 2024^57^ | Short-term learning effect of ChatGPT on pharmacy students' learning | Norway | Pharmacy | Both | ChatGPT. | Experimental randomised study - Pre-test and post-test knowledge tests, along with a questionnaire on knowledge and attitudes about ChatGPT. | To investigate the short-term learning effects of using ChatGPT among pharmacy students. | ChatGPT showed a potential to improve test scores, but no statistically significant effect was found in the short term​ | Production | Completing tasks and assignments | 13.5 |
| Weidener & Fischer, 2024^37^ | Artificial Intelligence in Medicine: Cross-Sectional Study Among Medical Students on Application, Education, and Ethical Aspects. | Germany, Austria, Switzerland | Medicine | Retrospective Survey/Interview on a Previously Administered Intervention | ChatGPT | Cross-sectional survey | To explore medical students' perceptions of AI applications, including ChatGPT, and the inclusion of AI and AI ethics in medical education. | The majority of students anticipated positive impacts from AI, but found current AI education insufficient, calling for the integration of AI ethics into their curricula | Inquiry, Practice, Production, Acquisition | Finding/ exploring information/data; Facilitating Critical Analysis, Problem-Solving, Decision-Making, and Refining Arguments; Assisting with communication and patient care; Assisting with performing clinical tasks; Completing tasks and assignments; Producing a written piece; Clarifying concepts and enhancing understanding | 9 |
| Worthing, Roberts and Šlapeta, 2024^60^ | Surveyed veterinary students in Australia find ChatGPT practical and relevant while expressing no concern about artificial intelligence replacing veterinarians | Australia | Veterinary | Retrospective Survey/Interview on a Previously Administered Intervention | ChatGPT | Cross-sectional survey | To assess veterinary students' perceptions of ChatGPT’s relevance and practicality in veterinary education and practice. | Students found ChatGPT practical and relevant for their studies, with no concerns about AI replacing veterinarians​ | Production | Completing tasks and assignments; Producing a writing piece | 8 |
| Wu et al., 2024^32^ | Application of ChatGPT-based blended medical teaching in clinical education of hepatobiliary surgery | China | Medicine | Empirical Research with an Active Intervention | ChatGPT | Prospective randomised controlled study | To evaluate the effectiveness of ChatGPT-based blended teaching in improving theoretical knowledge and clinical skills in hepatobiliary surgery | The ChatGPT-assisted group outperformed the control group in both theoretical exams and clinical skills assessments, with higher satisfaction and perceived teaching effectiveness​ | Inquiry, Practice, Acquisition | Finding/ exploring information/ data; Facilitating Critical Analysis, Problem-Solving, Decision-Making, and Refining Arguments; Researching; Assisting with communication and patient care; Assisting with performing clinical tasks; Revising materials | 12.5 |
| Zawiah et al., 2023^58^ | ChatGPT and Clinical Training: Perception, Concerns, and Practice of Pharm-D Students | Jordan | Pharmacy | Retrospective Survey/Interview on a Previously Administered Intervention | ChatGPT | Cross-sectional survey | To assess PharmD students' perceptions, concerns, and experiences regarding the integration of ChatGPT into clinical pharmacy training. | Students perceived benefits in using ChatGPT for clinical tasks but expressed concerns about over-reliance and accuracy, with varied levels of adoption in clinical training | Inquiry, Practice | Finding/ exploring information/ data; Assisting with communication and patient care; Assisting with performing clinical tasks; Seeking feedback and assisting with reflection | 9.5 |
